# NEM-Tar: A Probabilistic Graphical Model for Cancer Regulatory Network Inference and Prioritization of Potential Therapeutic Targets From Multi-Omics Data

**DOI:** 10.3389/fgene.2021.608042

**Published:** 2021-04-22

**Authors:** Yuchen Zhang, Lina Zhu, Xin Wang

**Affiliations:** ^1^Department of Biomedical Sciences, City University of Hong Kong, Hong Kong, China; ^2^Key Laboratory of Biochip Technology, Biotech and Health Centre, Shenzhen Research Institute, City University of Hong Kong, Shenzhen, China

**Keywords:** nested effects model, molecular subtype, regulatory network, drug targets, combination therapy, cancer

## Abstract

Targeted therapy has been widely adopted as an effective treatment strategy to battle against cancer. However, cancers are not single disease entities, but comprising multiple molecularly distinct subtypes, and the heterogeneity nature prevents precise selection of patients for optimized therapy. Dissecting cancer subtype-specific signaling pathways is crucial to pinpointing dysregulated genes for the prioritization of novel therapeutic targets. Nested effects models (NEMs) are a group of graphical models that encode subset relations between observed downstream effects under perturbations to upstream signaling genes, providing a prototype for mapping the inner workings of the cell. In this study, we developed NEM-Tar, which extends the original NEMs to predict drug targets by incorporating causal information of (epi)genetic aberrations for signaling pathway inference. An information theory-based score, weighted information gain (WIG), was proposed to assess the impact of signaling genes on a specific downstream biological process of interest. Subsequently, we conducted simulation studies to compare three inference methods and found that the greedy hill-climbing algorithm demonstrated the highest accuracy and robustness to noise. Furthermore, two case studies were conducted using multi-omics data for colorectal cancer (CRC) and gastric cancer (GC) in the TCGA database. Using NEM-Tar, we inferred signaling networks driving the poor-prognosis subtypes of CRC and GC, respectively. Our model prioritized not only potential individual drug targets such as HER2, for which FDA-approved inhibitors are available but also the combinations of multiple targets potentially useful for the design of combination therapies.

## Introduction

Cancers are always discovered with diverse molecular properties and heterogeneous clinical outcomes, even when occurring in the same tissues or organs. The last decade has witnessed tremendous progress in the emerging field of precision medicine for more accurate patient stratification for more optimized therapeutic treatment. However, it remains challenging to dissect the mechanism underlying cancer heterogeneity to identify novel drug targets for further development of targeted therapies. Targeted cancer therapy has been accepted as an effective weapon to conquer cancer (Green, 2004; Polyak and Garber, 2011), aiming to inhibit or reverse the activation patterns of particular cancer signaling pathways. Unfortunately, pathway redundancies, complex feedback, and crosstalk present in cancer cells often result in drug resistance, leading to treatment failure (Bernards, 2012; Yamaguchi et al., 2014). Therefore, a key task of precision medicine is excavating the causally wired relationship among the regulatory elements contributing to specific cancer molecular subtypes.

The identification of cancer therapeutic targets has long been based on biological knowledge and experience, which lacks a global functional overview and efficiency. Mathematical modeling could be established to predict potential drug targets in a more systematic and efficient way (Supplementary Table 1). Studies like iODA (Yu et al., 2020) integrated basic bioinformatic analysis and statistical methods to prioritize consistent molecular signatures at the pathway level for further investigation of cancer pathogenesis. Methods such as MiRNA-BD (Lin et al., 2018) focused on the discovery of novel miRNA biomarkers in diseases such as cancers without training or prior knowledge. Graphical models (e.g., Mezlini and Goldenberg, 2017; Manatakis et al., 2018; Kotiang and Eslami, 2020) were also proposed to infer the regulatory relationship and key driver genes, but the networks mainly encode gene expression associations, without support of multi-omics input. Other methods such as the miRNA-TF-mRNA network (Pham et al., 2019) and bipartite graphs (Bashashati et al., 2012) employed complex structures and multi-omics data to identify cancer driver genes as potential therapeutic targets. Furthermore, computational models were also proposed for the personalized prediction of potential target genes (Hou and Ma, 2014; Guo et al., 2018). All the previous methods have demonstrated their usefulness in various applications, very few of them infer causal regulatory relationships. To study the dysregulation of pathways and discover causal regulation relationships, typical approaches are Bayesian Networks, which encode conditional independence between genes on edges [e.g., (Sachs et al., 2005)]. However, the major limitation of Bayesian networks lies in their requirement of direct observations (e.g., protein activities) of perturbation effects on other pathway components, which are often not available. Besides, these methods require a large sample size to distinguish signal from noise and only capture parts of biologically relevant networks (Markowetz and Spang, 2007). Nested effects models (NEMs) (Markowetz et al., 2005, 2007) are specifically tailored to reconstruct signaling networks from indirect observations of experimental interventions. In each experiment, one component (e.g., kinase, transcription factor) in the pathway is perturbed, and multi-dimensional downstream effects are observed (e.g., gene expression or cell imaging data) (Siebourg-Polster et al., 2015). Different from other graphical models, NEMs encode subset relations between the observed downstream effects reporter genes under perturbations to signaling genes.

Nested effects models have been successfully applied to various biological scenarios to infer the causal network of signaling components (Markowetz et al., 2005; Fröhlich et al., 2009; MacNeil et al., 2015). Several extensions of NEMs have been proposed to adapt to different experimental designs or data types. For instance, Boolean NEMs (Pirkl et al., 2016) creatively model the data observed from arbitrary experimental combinations (excitation or inhibition) to infer a full Boolean network and further integrate the information from the literature. Epistatic NEMs (Pirkl et al., 2017) infer epistasis from phenotyping screens of double knock-downs systematically to test the hypothesis that complex relationships between a gene pair can be explained by the action of a third gene that modulates the interaction. Dynamic NEMs (Anchang et al., 2009; Fröhlich et al., 2011) infer the rate of the signal flow within the network from time-series data, while Hidden Markov NEMs (Wang et al., 2014) model the evolution of the network itself over time. Motivated by a recent experiment investigating epithelial-mesenchymal transition (EMT) in murine mammary gland cells, a method for mapping a non-interventional time series onto a static NEM has been proposed (Cardner et al., 2019). Furthermore, with the rapid development of single-cell sequencing technologies, a mixture of NEMs (M&NEM) tailored explicitly for single-cell data has been proposed (Pirkl and Beerenwinkel, 2018), which is capable of identifying different cellular subpopulations and inferring their corresponding causal networks simultaneously.

**FIGURE 1:**
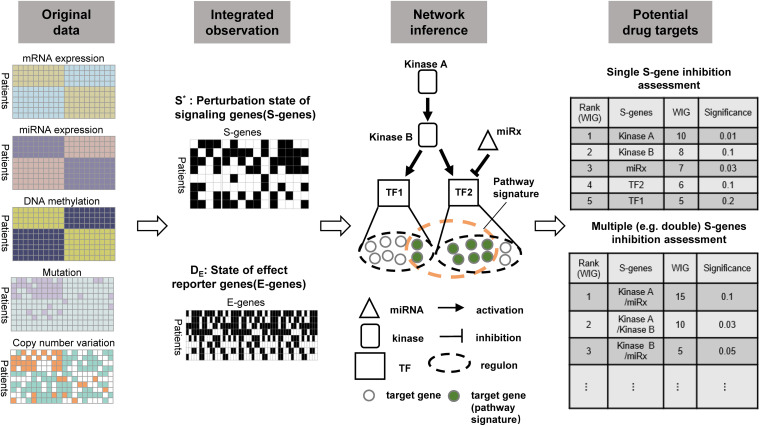
The workflow of NEM-Tar for cancer regulatory network inference and potential drug targets prioritization. Observations of the states of S-genes and E-genes could be obtained after the preprocessing of multi-omics data. The signaling network regulating a specific cancer subtype will subsequently be inferred. Finally, based on quantification of the causal impact and specificity to downstream genes using WIG, potential drug targets could be prioritized for single and double perturbations.

To prioritize potential therapeutic targets based on tissue-derived multi-omics profiles from cancer patients, we extended the classic NEMs to model the causal effects of genetic and epigenetic aberrations of various regulatory components (kinases, transcriptional factors, and miRNAs) on downstream genes. Importantly, the computational evaluation was conducted on the regulatory components (mainly on kinases) to prioritize potential therapeutic targets. Figure 1 illustrated the framework and major steps of NEM-Tar, which is featured with the following highlights: (1) Different from pre-existing NEMs developed for phenotyping screens derived from experimental perturbations, NEM-Tar integrates natural perturbations (e.g., somatic mutations, DNA hyper- or hypo-methylation, copy number alterations) at multiple levels of gene regulations for cancer-related signaling network inference; (2) We proposed a scoring method based on information theory, named weighted information gain (WIG), which could prioritize not only individual therapeutic targets but also evaluate potential combination therapies; (3) NEM-Tar is a versatile framework for dissecting the cancer molecular heterogeneity by inferring cancer subtype-specific signaling network. In our case studies, we specifically focused on the ‘EMT’ subtype in gastric cancer and the CMS4-mesenchymal subtype in colorectal cancer (Cristescu et al., 2015; Guinney et al., 2015), which are associated with a higher risk of recurrence and poor prognosis. Potential drug targets are evaluated specifically on the epithelial-mesenchymal transition (EMT) pathway, which is directly associated with cancer metastasis.

In the ‘Methods and Materials’ section, we introduce the design of NEM-Tar and the inference strategies in detail. Subsequently, we test the effectiveness of NEM-Tar in a simulation study (‘Results on Simulated Data’) and demonstrate its potential by real case studies on colorectal cancer and gastric cancer (‘Results on Case Studies’).

## Materials and Methods

### The Original Nested Effects Model (NEM)

We first review the original nested effect model (NEM), before we explain in detail how we extend the original model design to fit multi-omics high-throughput profiles of cancer samples.

**FIGURE 2:**
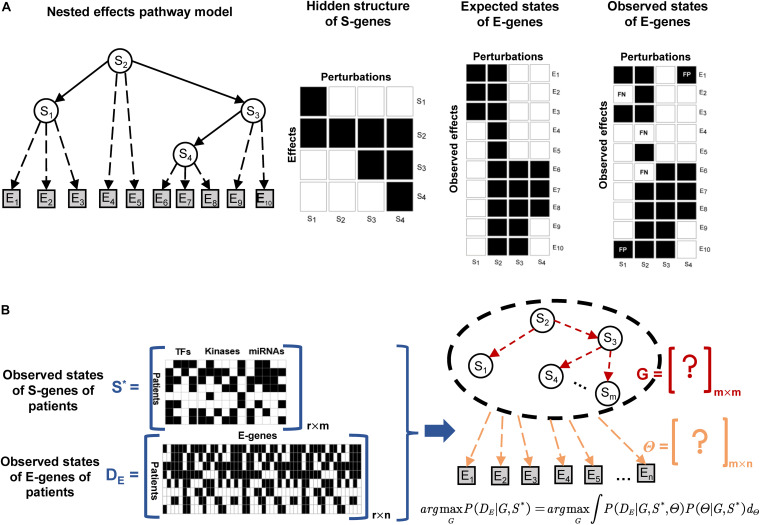
Illustration of the nested effects model and NEM-Tar for real cancer samples. **(A)** The S-genes are modeled as hidden variables, and their signaling interaction graph *G* (solid arrows) is the target to infer. In experiments with perturbations to individual S-genes, differential expression of downstream genes could be observed and considered as effect reporter genes (E-genes). Assuming that each E-gene is directly regulated by at most one S-gene in *G*, the maximum *a posteriori* attachment Θ (dashed arrows) of effect genes to S-genes could be computed. The goal is to search for the signaling graph *G*, which yields the most likely probabilistic nested effects. **(B)** For an extra observational dimension (the real patients), the necessary adjustment should be conducted on the design and inference strategies of classic NEM. However, the information that needs to be inferred is also the hidden interaction between S-genes and the attachment relationship of E-genes to S-genes.

The structure of a NEM is illustrated in Figure 2A. The goal is to infer a signaling network *G*, represented as a directed acyclic graph involving the regulators, also referred to as signaling genes (S-genes), denoted *S*_*j*_ for j∈{1,2,….,m}. In the initial phenotypic screening experiments, the S-genes are individually perturbed during RNAi experiments, but their effects are indirectly measured by the expression level of effect reporter genes (E-genes) denoted *E*_*i*_ for i∈{1,2,….,n}. The attachment of E-genes to S-genes is denoted by Θ, within which θ*_*ij*_* = 1, if E-gene *i* is attached to S-gene *j*. The initial NEMs assumed that each E-gene can be attached to at most one S-gene, but this constraint has been relaxed thereafter. Tresch and Markowetz have proposed to add a null S-gene, which predicts no effects to account for uninformative features (Tresch and Markowetz, 2008). Due to the nested effects, it is assumed that the signaling network *G* is transitively closed; for instance, in Figure 2A, the signaling information flow is S2→S3→S4, then S2→S4 also exists.

We calculate the expected E-gene profiles for a given model (*G;*Θ) as the matrix product with *E*_*ij*_ the predicted state of E-gene *i* under knock-down of S-gene *j*, namely *E*_*exp*_ = *G*Θ. In practice, we cannot neglect potential noise in the data, which requires probabilistic modeling to infer an optimal *G* to interpret the observation of E-genes. Suppose that we have a candidate network structure *G*, which is a directed acyclic graph (DAG) of S-genes. What matters ultimately is the posterior probability of the model:

(1) $$P\,\left(G|E\right)=\frac{P\,\left(E|G\right)\,P\,\left(G\right)}{P\,\left(E\right)}$$

where the denominator does not depend on *G* and cannot be taken into consideration for model comparison. Since almost nothing is known about the signaling network without reliable knowledge, we use a uniform prior P(G). Thus, we focus entirely on the likelihood P(E|G). It can be computed by marginalizing over E-gene attachments Θ, or by employing the maximum *a posteriori* (MAP) estimate of Θ (Tresch and Markowetz, 2008). The former choice is more intuitive, and the marginal likelihood can be deduced as:

(2) $$P\,\left(G|E\right)=\int P\,\left(E|G,\Theta \right)\,P\,\left(\Theta ,G\right)\,d\Theta =\frac{1}{m^{n}}\prod_{i=1}^{n}\sum_{j=1}^{m}\prod_{k=1}^{l}P\left(e_{i\,k}|G,\theta_{i}=j\right)$$

The term *P*(*e*_*ik*_|*G*,θ_*i*_ = *j*) in Eq. 2 reflects the noise rate of the real binary observation *e_*ik*_.* The distribution of *e*_*ik*_ is determined by the network structure *G* and the error probabilities α and β. For all E-genes and targets of perturbation, the conditional probability of the E-gene state *e*_*ik*_ given the network structure *G* can then be written as:

(3) $$P\left(e_{i\,k}|G,\theta_{i}=j\right)=\begin{array}{c} {}^{e_{i\,k}=1\;e_{i\,k=0}}\\ \left\{ \begin{array}{cc} \alpha \; & 1-\alpha \\ 1-\beta \; & \beta \end{array}\right. \end{array}$$

Equation 3 means that if *E*_*i*_ is not an influenced target of the S-gene perturbed in experiment *k*, the probability of observing *e_*ik*_* = 1 is α (probability of false alarm, type-I error); the probability to miss an effect and observe *e_*ik*_* = 0 even though *E*_*i*_ is an influenced target is β (type-II error).

### NEM-Tar for Multi-Omics Data

Figure 1 illustrated the NEM-Tar framework and the major steps involved to infer a signaling network using a toy example, and a comparison was made with a classic NEM in observation (Figure 2B). We model copy number variations or mutations (e.g., copy number gain/mutation in kinase A/B, mutation in transcription factor TF1), hyper/hypo methylation (e.g., hypermethylation of miRx) as ‘natural’ perturbations in tumors, which are different from experimental perturbations such as RNA interference and CRISPR-Cas9 knockout modeled in the classic NEMs. Regulators considered in the network are master regulators (TFs and miRNAs) and modulators (kinases) resulting from the reported literature (Fessler et al., 2016; Kiyozumi et al., 2018; Xie et al., 2020) and our prioritized candidates. Let *S*^∗^ = [*s*_*kj*_] denote the state matrix of regulators, where *s*_*kj*_ represents whether regulator *j* is aberrant in sample *k* or not. Let *G* represent the signaling network of interactions between kinases, TFs, and miRNAs, and Θ be the set of interactions between regulators and their target genes. Let *D* = [*e*_*ki*_] be the observed data, where *e*_*ki*_ denotes whether the E-gene *i* is differentially expressed in patient *k* (*e*_*ki*_ = 1) or not (*e_*ki*_* = 0). Our goal is to infer the optimal *G* that maximizes the following marginal likelihood:

(4) $$\arg\max_{G}P\,\left(D|G,S^{*}\right)=\arg\max_{G}\,\int P\,\left(D_{E}|G,S^{*},\Theta \right)\,P\,\left(\Theta |G,S^{*}\right)\,d\Theta$$

It should be noted that Eq 4 is similar to the original likelihood function of NEMs (Eq. 2), except the state matrix of regulators (S-genes) in our model.

When the optimal S-genes structure *G*^∗^ is determined, we could compute the posterior probability for the edge between *S*_*j*_ and *E_*i*_.*

(5) $$P\left(\theta_{i}=j|G^{{}^{*}},S^{*},D\right)=\frac{1}{Z}\prod_{k=1}^{l}P\left(e_{k\,i}|G^{*},S^{*},\theta_{i}=j\right)$$

where Z is a constant and does not rely on *G*^∗^.

When using NEM-Tar in real-world applications, we recommend the following criteria to select E-genes and S-genes. E-genes can be prioritized based on genes that are significantly upregulated (log_2_FC > 1, FDR < 0.01) in a specific cancer subtype of interest. If the selected E-genes are too few (e.g., only 238 E-genes for the EMT subtype of GC based on the above criteria), the cutoff on log_2_FC may be relaxed to 0.5. The prioritization of S-genes can be based on the following criteria. First, subtype-specific miRNAs and TFs can be prioritized based on differentially expressed genes. By default, we recommend selecting TFs that are significantly upregulated (log_2_FC > 1 and FDR < 0.01) and miRNAs that are significantly downregulated (log_2_FC < −1 and FDR < 0.01). However, due to the heterogeneity between different cancer subtypes, the number of candidate miRNAs or TFs may be limited. In the situation, the cutoff on log_2_FC may also be relaxed to 0.5. Second, the selection of S-genes should also satisfy the following perturbation criteria: (1) Mutation: the cutoff on mutation frequency in kinases/TFs should be >5%. When the overall mutation frequency of candidate S-genes is lower than 5%, the cutoff might also be relaxed appropriately. (2) copy number variations (CNVs): kinases and membrane proteins with >5% frequency of copy number gains. (3) DNA methylation: miRNAs with significant hypermethylation (delta-beta >0.1, BH-adjusted *P* < 0.001).

### Inference Methods of NEM-Tar

The original NEM performs an exhaustive search over all transitively closed graphs to identify the optimal graph by the maximum likelihood estimation (Markowetz et al., 2005). Since the number of candidate network structure *G* grows exponentially with the number of nodes, an exhaustive enumeration is not feasible for signaling networks with more than five S-genes. In real applications, it is always necessary to search for a larger network, where heuristics are more appropriate to explore the network space. Many heuristic inference methods have been proposed, with respective advantages as well as limitations. To determine the optimal inference strategy for NEM-Tar, we investigated the triple relations, greedy hill-climbing, and MCMC sampling methods.

Instead of scoring the whole network, the model could be learned using a pairwise method (Markowetz et al., 2007). For a pair of genes *A* and *B*, their relationship could be determined by maximum *a posteriori* (MAP) from four possible models: *A*⋅*B* (unconnected), *A*→*B* (effects of A are a superset of effects of B), *A* ← *B* (subset), and *A* ↔ *B* (undistinguishable effects). However, the pairwise learning assumes independence of edges, which is not true in transitively closed graphs. Hence, the natural extension of pairwise learning is the inference from the triples of nodes (Markowetz et al., 2007), which comprises two steps. First, for each triple (*x,y,z*) in the graph with *n* nodes, all 29 possible quasi-orders are scored, and the MAP model is selected. Edgewise model averaging was subsequently employed to combine all models into the final graph.

Greedy hill-climbing is a more straightforward optimization strategy known from the literature (Russell and Norvig, 2016). Given an initial network hypothesis (usually an empty graph), a local maximum of the likelihood function could be reached by successively adding an edge. This procedure is continued until no improving edge can be found anymore. We also evaluated the performance of greedy hill-climbing for the benchmark in our simulation study.

Furthermore, Markov chain Monte Carlo (MCMC) methods are a class of algorithms for sampling from a probability distribution. Niederberger et al. (2012) proposed an inference method by combining MCMC sampling with an Expectation-Maximization (EM) algorithm. For reconstructing evolving signaling networks, MCMC sampling was also an important procedure in HM-NEM (Wang et al., 2014). In our simulation study, we also examined MCMC sampling, and the detailed pseudocode is in Supplementary Figure 1.

### Weighted Information Gain (WIG) for Evaluation of the Causal Impact of S-Genes on Downstream Reporter Genes

Given the inferred optimal network *G*^∗^ and interactions between regulators and target genes Θ^∗^, we sought to quantify the causal impact that a regulator has on downstream reporter genes, especially signature genes for a particular biological process of interest such as epithelial–mesenchymal transition (or EMT) (Nieto et al., 2016; Lambert et al., 2017). The fundamental assumptions of the assessment criteria for the impact should satisfy: (1) The more E-genes related to a particular pathway are affected by an S-gene, the more significant the influence is; (2) The more likely a particular E-gene is attached to an S-gene, the higher the global influence of the S-gene is. On the basis of the above assumptions, we defined a score called **Weighted Information Gain (WIG)** on every E-gene within the regulons of S-genes based on KL divergence (Kullback and Leibler, 1951) in information theory, which measures the information gain after network inference.

(6) $$WIG({\text{S}}_{j})=\Sigma_{i=1}^{r}WIG(S_{j}\to E_{i})=\Sigma_{i=1}^{r}P(S_{j}\to E_{i})\log[(m+1)P(S_{j}\to E_{i})]$$

**FIGURE 3:**
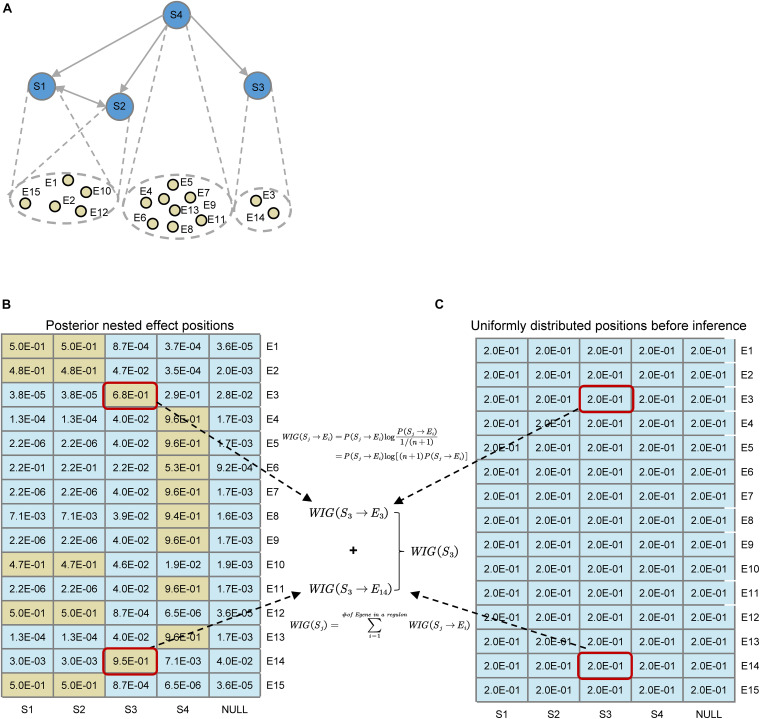
Illustration for the definition of Weighted Information Gain (WIG) using a toy example. **(A)** A toy network containing four S-genes with their corresponding E-gene attachment. Note that the hierarchies of S1 and S2 genes cannot be distinguished. **(B)** Posterior effect positions obtained after network inference; **(C)** Uniformly distributed effect positions before inference. Suppose that the attached E-genes to a S-gene are all signature genes related to a pathway of interest (e.g., EMT), it could be easily calculated that S_4_ has the highest causal impact on the particular downstream pathway, and S_1_ and S_2_ have the same impact. As an example, we illustrated the calculation of *WIG*(S_3_).

As shown in Figure 3, before the network inference, for every E-gene, we assume that the probability of an E-gene attached to an S-gene is uniformly distributed, which could be denoted as*P*(θ_*i*_ = *j*|*G*) = 1/*m* + 1, if we set a ‘null’ S-gene and no particular prior knowledge is involved. While after the inference, the posterior distribution of nested effect positions of E-genes changes into*P*(*S*_*j*_→*E*_*i*_) = *p*(θ_*i*_ = *j*|*G*^∗^,*S*^∗^,*D*). According to the original definition of KL divergence, the increase of the information of the attachment of an E-gene could be computed, like the highlighted *WIG*(*S_3_→E_3_*) and *WIG*(*S_3_→E_14_*). As for an S-gene, the global causal impact over all the E-genes or some signature genes of key pathways could be obtained by summing up the WIG of related E-genes, as shown in Eq. 6. The statistical significance for the specificity of WIG on key pathways could be estimated by the bootstrap of the same number as the pathway signature genes of arbitrary E-genes within the regulon of a S-gene.

Ultimately, kinases/TFs/miRNAs with top causal WIG and/or enough significance will be prioritized as potential drug targets. For more convenient drug design, kinases, or membrane proteins are preferred.

### Data Source

In our case studies, we analyzed multi-omics data for colorectal cancer (CRC) and gastric cancer (GC) patients from TCGA, including the following data types: (1) whole-genome gene expression data for 382 CRC and 415 GC patients based on RNA sequencing platform; (2) copy number variation data (scores on gene level) for 374 CRC patients and 268 GC patients; (3) somatic mutations profiles for 423 CRC patients and 433 GC patients; (4) miRNA expression data for 297 CRC and 446 gastric tumors based on Illumina sequencing platform; (5) DNA methylation data for 396 CRC and 395 GC tumor samples based on Infinium Methylation 450K platform.

## Results

### Results on Simulated Data

#### Generation of *in silico* Data

The simulations evaluating the inference strategies of NEM-Tar were performed on datasets generated with varying network sizes and noise levels. The generation of simulated data is described in detail as follows.

(1) **S-gene graph generation**: We first randomly generated a graph of *m* S-genes, m∈ {6,8,10,12,15,20,30}. These graphs of S-genes were transformed to transitively closed graphs.

(2) **S-gene state generation**: For each S-gene graph generated, we simulated patient samples with a random fraction of S-genes perturbed according to the real proportions of S-genes with genetic and epigenetic alterations in the gastric cancer case study. An S-gene state matrix was subsequently generated according to the S-gene graph and simulated perturbations.

(3) **Attachment of E-genes to S-genes**: In each S-gene graph simulated, we attached effect reporter genes (or E-genes) to each S-gene, and the number of E-genes per S-gene was roughly equivalent to the average number of E-genes in the gastric cancer case study.

(4) **Generation of E-gene observations**: For each simulated graph, with the corresponding S-gene state matrix and E-gene attachment, we next generated the corresponding E-gene observation matrix. For E-genes without downstream effects expected, observations were sampled from a null distribution, or otherwise from an alternative distribution. In the simplest case, we only sampled binary data, where 1 indicated an effect and 0 no effect, according to the Type-I error α_*sim*_ (FP) and Type-II error β_*sim*_ (FN).

Using the simulation strategy, we generated data to test the performance of NEM-Tar:

(1) Scalability. Fix α_*sim*_ = β_*sim*_ = 0.05 and vary the number of S-genes from 6 to 30, representing the size of a typical signaling pathway. For each number of S-genes, 200 different random S-gene networks were generated, and the simulated S-gene network structures were inferred using MCMC sampling, triple relations, and greedy hill-climbing, respectively;

(2) Robustness to noise. Fix β_*sim*_ = 0.05 and the number of S-genes *m* = 12 (medium size) and vary α_*sim*_ from 0.05 to 0.5. For the inference of S-gene network, we set α = 0.2, β = 0.1, which were arbitrarily chosen and different from the α_*sim*_ and β_*sim*_ used for the generation of E-gene data. The evaluation criteria of their performance were TPR = TP/(TP + FN), TNR = TN/(TN + FP), Accuracy = (TP + TN)/(TP + FN + TN + FP) and Precision = TP/(TP + FP).

#### Benchmark the Performance of Inference Methods

**FIGURE 4:**
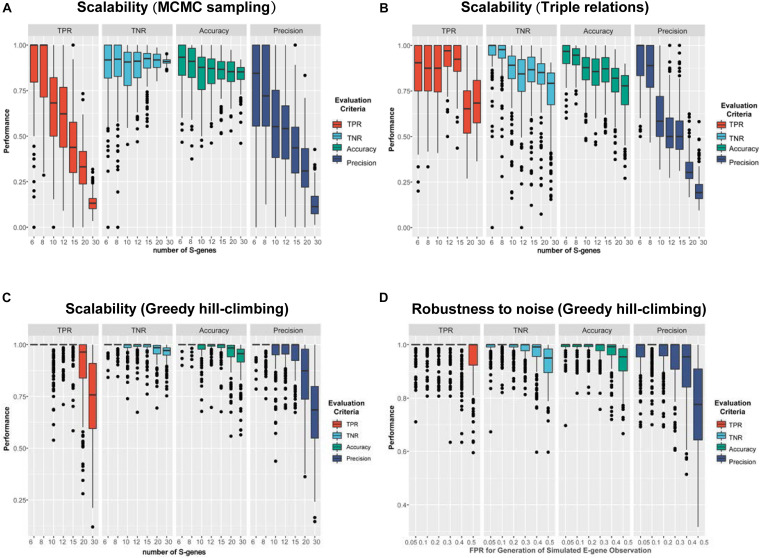
A comparison of the performance of three representative network inference strategies. **(A–C)** The performance of NEM-Tar based on **(A)** MCMC sampling, **(B)** triple relations, and **(C)** greedy hill-climbing, respectively, on simulated data for varying numbers of S-genes. For each method, we generated 200 random signaling networks and inferred their structures using NEM-Tar from the simulated E-gene data. **(D)** The performance of NEM-Tar based on greedy hill-climbing testing its robustness to simulated data with different levels of noise.

The simulation results are shown in Figure 4. Using MCMC sampling (Figure 4A), although the performance showed a decreasing trend due to the increase of the size of the network, the magnitude of decrease was quite significant (e.g., the averaged TPR of 200 networks decreased from 0.867 to 0.136). Especially, the most concerned measure ‘Precision’ was unacceptable in real applications, no matter for smaller networks (S-genes ≤ 10) or larger networks (S-genes > 10). Even for smaller networks with only six S-genes the instability of MCMC was evident, as the median of Precision (0.845) was much larger than the mean (0.770). For relatively large networks (e.g., 20 S-genes), the averaged Precision was too low (0.328) to accept. Using the triple relations inference, the result was slightly better than MCMC sampling (Figure 4B), but a dramatic decrease of Precision was also observed for networks with ≥ 10 S-genes. A special observation on the triple relations is that the performance on the networks with a medium size (10-15) showed fluctuating TPR, TNR, and Accuracy rather than a steady decrease, suggesting that the inference based on triple relations was also unstable.

Compared to MCMC sampling and triple relations methods, greedy hill-climbing showed much higher performance (Figure 4C-D). For small and medium networks (6-15 S-genes), the median of all the evaluation metrics were close to 1. Even for relatively large networks, the TPR and Precision were still reliable. Though, in essence, the greedy hill-climbing is likely to be trapped in a local optimum, at least for the graphs with less than 30 S-genes, the performance is reasonably good. The robustness for the inference with varying α_*sim*_ based on greedy hill-climbing is also stable and acceptable. Even for the very noisy condition (α_*sim*_ = 0.5), the averaged TPR and Precision could still reach 0.947 and 0.766, respectively. Furthermore, compared to the other two methods, the greedy hill-climbing algorithm was not only superior in the performance, but also less time consuming (Supplementary Table 2).

Therefore, based on the simulation study, we employed greedy hill-climbing as the inference method for the following case studies.

### Results on Case Studies

To exemplify NEM-Tar for inference of cancer subtype-specific signaling network and prioritization of potential therapeutic targets, we did two case studies using multi-omics data in gastric cancer and colorectal cancer, respectively.

#### Inferring the Signaling Network Driving the EMT Subtype of Gastric Cancer and Prioritization of Potential Drug Targets

Gastric cancer (GC), a leading cause of cancer-related deaths, is known to be a heterogeneous disease. The presence of molecular heterogeneity in GC has been shown through the existence of subtypes with distinct genetic/epigenetic aberrations associated with clinical outcomes. Based on 300 primary gastric cancer tumor specimens, the Asian Cancer Research Group (ACRG) identified four molecular subtypes with distinct patterns of molecular alterations and clinical outcomes (Cristescu et al., 2015). Among these four subtypes, patients classified to the MSS/EMT (in short, EMT) subtype showed the worst prognosis. Despite the extensive subtyping studies published, the regulatory mechanism underlying specific molecular subtypes has not been fully explored explicitly. Here, we employed NEM-Tar to infer the signaling network driving the EMT gastric cancer and quantitatively evaluate single and double perturbations to prioritize potential drug targets.

For the choice of the regulatory elements, we focused on the signature genes of the MAP-kinase pathway (KRAS, BRAF), frequently mutated kinases/TFs (TP53, ARID1A, CDH1, and ERBB2) (Gastric Adenocarcinoma - My Cancer Genome) and significantly upregulated TFs (log_2_FC > 0.5, BH-adjusted *P* < 0.01) as well as downregulated miRNAs (log_2_FC < −1, BH-adjusted *P* < 0.01) in the EMT subtype. The regulatory elements were filtered through the integration with the somatic mutation profiles. More specifically, we kept the kinases and TFs with mutation frequency > 5% and ZEB2 (Dai et al., 2012) and KRAS (Yoon et al., 2019), which were well characterized before for their roles in EMT regulation. As a result, we included nine kinases/TFs in 177 patient samples for the following analysis. The perturbations to miRNAs were measured by DNA methylation in the promoters, and six miRNAs were selected with highly significant hypermethylation (delta-beta > 0.1, BH-adjusted *P* < 0.001) in the samples of the EMT subtype. Since copy number variations (CNVs) were frequently found in kinases and membrane proteins in many cancer types, we also incorporated copy number gains as a type of perturbation in the case study. Furthermore, 1194 genes significantly upregulated in the EMT subtype (log_2_FC > 0.5, BH-adjusted *P* < 0.01) were selected as E-genes for the following analysis.

In the classic NEMs, E-genes’ states are the production of individually perturbed S-genes, while for NEM-Tar E-genes’ states can be the production of multiple S-genes with genetic and/or epigenetic perturbations in a tumor sample. Therefore, the concepts of positive and negative controls for the discretization of E-genes’ states should be revised accordingly. A positive control referred to the patients belonging to a particular subtype (e.g., EMT subtype) but without any (epi)genetic aberrations in the S-genes. In contrast, a negative control referred to patients not assigned to a particular subtype (e.g., Non-EMT subtypes) and had no aberrations in any S-genes. Using the strategy, we transformed the continuous gene expression data into binary observations. Denote *C*_*ik*_ as the continuous expression level of *E*_*i*_ of patient *k*. Let μ*_*i*_*^+^ be the mean of positive controls for *E*_*i*,_ and μ*_*i*_*^–^ the mean of negative controls. To derive binary data *E*_*ik*,_ we defined individual cutoffs for every gene *E*_*i*_ by:

(7) $$E_{ik}=\left\{ \begin{array}{l} 1\;if\;C_{ik}<\sigma .{\text{μ}}_{i}^{+}+(1-\sigma ).{\text{μ}}_{i}^{-}\\ 0\;else \end{array}\right.$$

Based on the method introduced in Markowetz et al. (2005), to make a balance between Type-I and Type-II errors, we set σ = 0.5 for the discretization. As a result, we obtained the estimated error rates α = 0.07, β = 0.08.

**FIGURE 5:**
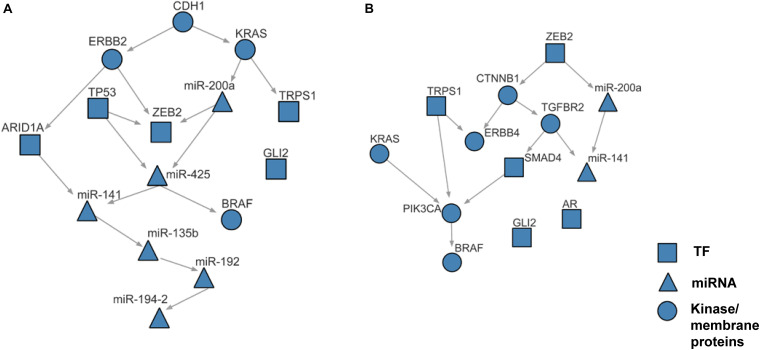
The case studies of NEM-Tar on gastric cancer and colorectal cancer. **(A)** Reconstructed signaling network for the EMT subtype of gastric cancer. **(B)** Reconstructed signaling network for the CMS4 subtype of colorectal cancer.

Using the discretized E-gene data, we inferred the S-gene network regulating the EMT subtype of GC using NEM-Tar (Figure 5A). Interestingly, CDH1, ERBB2 (HER2), and KRAS were predicted to be sitting at the top hierarchies in the signaling network. Indeed, Trastuzumab, a monoclonal antibody for human epidermal growth factor receptor 2 (HER2), has already been established with chemotherapy as a first-line treatment for HER2-positive metastatic advanced GC patients (Bang et al., 2010). Besides, CDH1, coding for the E-cadherin protein, was reported to be linked to GC susceptibility and tumor invasion, and preliminary studies indicated the potential clinical value to employ CDH1 haplotypes in metastatic GC to stratify patients that will benefit from Trastuzumab-based treatments (Caggiari et al., 2017). NEM-Tar further supports the important discovery by computationally predicting and statistically evaluating the potential drug targets. Summarizing the single and double S-gene perturbations (to kinases only) with top WIGs, we found that both CDH1 and HER2 had a strong causal impact on the signature genes of epithelial-mesenchymal transition (EMT) (Zhao et al., 2019). More importantly, the causal effect was statistically significant and specific to the EMT pathway only (Table 1), as quantified by permutation tests, i.e., random sampling of E-genes with the same number of EMT signature genes in the regulon of a S-gene, and calculating the frequency of observing a same or higher WIG from the sampled E-gene sequences. Moreover, the combinatorial perturbations (Table 2) to CDH1 and ERBB2, CDH1 and KRAS or CDH1 and BRAF had the strongest and specific causal effect on the EMT pathway among all possible combinations.

**TABLE 1:** WIGs assessing the impact of single perturbations (kinase only) on EMT in GC.

S-genes	Total No. of downstream E-genes within the regulon	No. of E-genes (EMT related) within the regulon	WIG	Significance of WIG (100,000 sampling, BH-adjusted *P*)
CDH1	591	57	66.15	<1e-05
ERBB2	491	44	51.65	<1e-05
KRAS	229	20	20.05	<1e-05
BRAF	14	1	2.71	3.21e-01

**TABLE 2:** Double perturbations (kinase only) with top WIGs in GC.

S-genes	Total No. of downstream E-genes within the regulon	No. of E-genes (EMT related) within the regulon	WIG	Significance of WIG (50,000 sampling, BH-adjusted *P*)
CDH1/ERBB2	591	57	66.15	<5e-04
CDH1/KRAS	591	57	66.15	<5e-04
BRAF/CDH1	591	57	66.15	<5e-04
KRAS/ERBB2	558	51	59.12	<5e-04
BRAF/ERBB2	505	45	54.36	<5e-04
KRAS/BRAF	229	20	20.05	<5e-04

#### Inferring the Signaling Network Driving the CMS4-Mesenchymal Subtype of Colorectal Cancer and Prioritization of Potential Drug Targets

Similar to gastric cancer, colorectal cancer (CRC) is also a heterogeneous disease posing a challenge for accurate classification and treatment of this malignancy. Recently, CRC patients have been categorized using unsupervised classification of gene expression profiling, which resulted in distinct CRC subtypes. In order to generate unified subtyping of CRC, based on a large panel of CRC patients (*n* = 4151), the CRC Subtyping Consortium identified four consensus molecular subtypes (CMSs) (Guinney et al., 2015). Linking the subtypes to disease outcomes revealed that the mesenchymal subtype CMS4 displayed a worse prognosis, highlighting the clinical relevance of the CMS taxonomy. As another case study, we employed NEM-Tar to infer the signaling network driving the CMS4 CRC and calculated WIGs for single and double perturbations to signaling elements in order to prioritize potential drug targets.

To select regulatory elements, we incorporated the signature genes of the MAP-kinase pathway (KRAS, BRAF, PIK3CA), and the TFs significantly upregulated in CMS4 (log_2_FC > 1, BH-adjusted *P* < 0.01) as well as the miRNAs significantly downregulated in CMS4 (log_2_FC < −0.5, BH-adjusted *P* < 0.01). The regulatory elements were filtered through the integration with the somatic mutation profiles. More specifically, the kinases and TFs with the mutation frequency > 5% were left, resulting in 11 kinases/TFs in 212 patient samples for analysis. The perturbations to miRNAs were measured by DNA methylation in the promoters, and two miRNAs were selected with highly significant hypermethylation (delta-beta > 0.1, BH-adjusted *P* < 0.001) in the samples of the CMS4 subtype. The copy number variations (CNVs) profiles were also preprocessed, but the frequency of copy number gain was too low (less than 5%) to integrate. Finally, after integration with downstream E-genes (log_2_FC = 1, BH-adjusted *P* = 0.01) that are differentially expressed between CMS4 and non-CMS4 samples, we obtained a 212 × 1337 E-gene observation matrix for the following analysis.

The whole discretization analysis of E-genes is similar to what we did in gastric cancer. In CRC, the positive controls are patients belonging to the CMS4 subtype without any aberrations in any S-genes, while the negative controls are patients assigned to Non-CMS4 subtypes without aberrations in any S-genes. We set σ = 0.6 for the discretization, and the estimated error rates were α = 0.22 and β = 0.18. Using the discretized E-gene data, we inferred the S-genes network regulating the CMS4 subtype of CRC (Figure 5B). Based on the WIG calculation (Table 3, 4), we found that the perturbation on KRAS has the highest impact on the EMT pathway, though the influence is not specific to EMT, which is reasonable as KRAS is a frequently mutated oncogene in cancer. Currently, a variety of methods to inhibit KRAS for the treatment of metastatic CRC have been proposed (Porru et al., 2018). Besides, CTNNB1, which encodes β-catenin, has the second highest impact on the EMT pathway. CTNNB1 is involved in the Wnt-β-catenin signaling pathway, which often drives a transcriptional program that is reminiscent of EMT (Anastas and Moon, 2013). Particularly, the role of Wnt-β-catenin signaling in CRC and its potential as a therapeutic target for CRC has been extensively explored. Existing drugs targeting β-catenin, such as Aspirin, are already available, and several small molecules are under clinical trials (Cheng et al., 2019). Furthermore, the combinatorial perturbations to KRAS and CTNNB1, as well as KRAS and TGFBR2, enhanced the causal impact on the EMT pathway compared to their single perturbations, suggesting potential combination therapies for the specific CMS4 subtype of CRC.

**TABLE 3:** WIGs assessing the impact of single perturbations (kinase only) on EMT in CRC.

S-genes	Total No. of downstream E-genes within the regulon	No. of E-genes (EMT related) within the regulon	WIG	Significance of WIG (100,000 sampling, BH-adjusted *P*)
KRAS	525	49	38.61	1.48e-01
CTNNB1	151	14	23.26	< 1e-05
TGFBR2	85	10	16.67	< 1e-05
PIK3CA	26	3	5.98	3.14e-02
BRAF	15	2	3.94	5.21e-01
ERBB4	23	2	2.95	5.25e-01

**TABLE 4:** Double perturbations (kinase only) with top WIGs in CRC.

S-genes	Total No. of downstream E-genes within the regulon	No. of E-genes (EMT related) within the regulon	WIG	Significance of WIG (50,000 sampling, BH-adjusted *P*)
KRAS/CTNNB1	650	60	55.88	<5e-04
KRAS/TGFBR2	584	56	49.29	2.73e-04
KRAS/ERBB4	548	51	41.56	1.06e-01
KRAS/BRAF	525	49	38.61	2.62e-01
KRAS/PIK3CA	525	49	38.61	1.71e-01
BRAF/CTNNB1	151	14	23.26	<5e-04
PIK3CA/CTNNB1	151	14	23.26	<5e-04
TGFBR2/CTNNB1	151	14	23.26	<5e-04
ERBB4/CTNNB1	151	14	23.26	< 5e-04
TGFBR2/ERBB4	108	12	19.62	4.20e-05

## Discussion

Although quite a few computational approaches have been developed for the identification of cancer therapeutic targets, they differ in the types of input data, the design of models/algorithms, the output of the results and the angles of biological interpretations. The unique strength of our NEM-Tar lies in its capability to prioritize not only individual therapeutic targets but also combinational therapies, which has not been realized before as far as we know. As a result, it is very difficult to quantitatively compare NEM-Tar with other computational approaches directly. However, we tried to make a rough comparison with two widely used methods, DawnRank (Hou and Ma, 2014) and DriverNet (Bashashati et al., 2012), which were proposed to discover cancer driver genes. Using DawnRank, we found that for the CMS4 subtype in CRC, AR, and GLI2, two TFs in our regulatory network, were also ranked among the top 5% (Supplementary Table 3). More excitingly, CDH1 and TP53 were ranked as the top two drivers for the EMT subtype in GC (Supplementary Table 3). When it comes to the result of DriverNet, only CDH1 and TP53 were prioritized as the 2nd and 3rd for EMT subtype in GC (Supplementary Table 4). However, no driver genes were found consistent between NEM-Tar and DriverNet for the CMS4 subtype of CRC (Supplementary Table 5). It should be noted that DawnRank and DriverNet could dissect the driver genes only based on the modeling of association networks, which lack the inference of causal relationships and cannot measure double or multiple therapeutic targets. Furthermore, neither DriverNet nor DawnRank were designed to distinguish TFs and kinases and could not incorporate perturbation information at other levels of gene expression regulations except for gene mutations. Instead, NEM-Tar was developed to prioritize potential therapeutic targets using regulatory network inference based on nested effects models.

The hierarchical causal relationship between signaling components is not only central for understanding the regulatory mechanism of cancers but also critical for developing potential drug targets to overcome the pervasive genetic redundancies. Inspired by NEMs encoding subset relations between observed downstream effects of experimental perturbations in signaling genes, we proposed NEM-Tar to infer signaling networks from various genetic and epigenetic perturbations to regulatory elements such as kinases, transcriptional factors, and miRNAs. The marginal likelihood function of NEM-Tar is similar to the original likelihood function of NEM, except the state matrix of regulators (S-genes) in our model. Based on NEM-Tar, a new score named weighted information gain (WIG) was defined to assess the causal impact of S-genes on downstream reporter genes.

Colorectal cancer and GC are two major malignancies of the gastrointestinal tract, for which molecular subtyping has been well studied. To exemplify the usefulness of NEM-Tar, we performed two case studies to infer signaling networks that drive the poor prognosis subtypes of GC and CRC, respectively. In GC, we found that among the top significant signaling genes with high WIGs, CDH1, and ERBB2 are particularly attractive. Indeed, the FDA-approved drug Trastuzumab targeting ERBB2 has already been established with chemotherapy as a first-line treatment for HER2-positive metastatic advanced GC patients. Our further evaluation of combinatorial perturbations suggested that simultaneous inhibition of CDH1 and ERBB2/KRAS/BRAF, ERBB2, and KRAS/BRAF, as well as KRAS and BRAF may be potential combination therapies. For CMS4 CRC, except for KRAS, a representative oncogene employed as a therapeutic target, the kinase CTNNB1 with the second highest WIG may be a potential alternative therapeutic target to CRC, and combinatorial inhibition of KRAS and CTNNB1 may provide a potential combination therapy.

Within the inferred signaling networks, we noticed many interesting interactions between the S-gene regulators. First, in the signaling network inferred for the EMT subtype in GC (Figure 5A), CDH1 and ERBB2 were prioritized as potential therapeutic targets (Table 1). A signal flow was inferred between them, which could be explained by the direct interaction (PPI) between them (Guo et al., 2014) or their PPIs via β-catenin (CTNNB1) (Schroeder et al., 2002; Tang et al., 2008). The signal flow miR-200a→ZEB2 could be strongly supported by the previous finding that miR-200a can regulate the expression of ZEB2 by directly binding the 3′UTR (Cong et al., 2013). Furthermore, the signal flow KRAS→miR-200a was also supported by the previous finding that oncogenic KRAS activation can suppress the expression of miR-200s (Zhong et al., 2016), and TP53→ZEB2 could be verified by their interactions with the miR-200 family (Rokavec et al., 2014). Second, in the signaling network inferred for the CMS4 subtype in CRC (Figure 5B), the advantages of our work were demonstrated more explicitly. The signal flow KRAS→PIK3CA→BRAF, supported by the MAP-kinase pathway (Dhillon et al., 2007), i.e., the PPIs between KRAS and PIK3CA (Hart et al., 2015) and between PIK3CA and BRAF (Shen et al., 2017), which is known as a typical signaling pathway driving EMT. The interaction between TGFBR2 and SMAD4 is involved in the TGFβ signaling pathway (Zhang et al., 1996). The signal flow CTNNB1→TGFBR2 is involved in the crosstalk between Wnt/β-catenin and TGFβ signaling pathways (Tian and Phillips, 2002). Together, the literature supports the effectiveness of NEM-Tar in predicting the regulatory hierarchy involving multiple redundant pathways driving EMT. Moreover, we also found signal flows between miRNAs, like the links miR-200a→miR-425, miR-141→miR-135b (Figure 5A) and miR-200a→miR-141 (Figure 5B), which are interesting but have not been previously reported yet. The miRNAs may interact indirectly via intermediate regulators, which were not included in the regulatory network inference based on our criteria for the selection of S-genes. The crosstalk between the miRNAs might also indicate their synergistic relationship on co-regulating downstream targets, which is frequently reported in the literature [reviewed in Xu et al. (2016)]. Integrating the computational prediction with experimental validation will be more convincing in revealing the crosstalks between the miRNAs, which will be an interesting direction to explore in our future work.

NEM-Tar can be improved in multiple ways in our future work. First, known signaling pathway structures can be incorporated into the model as prior knowledge to strengthen the accuracy of inference. Second, NEM-Tar proposed in this article is designed for binary effects and treats E-genes as independent random variables. However, we can possibly model log odds ratios like the methods in Tresch and Markowetz (2008), where alternative and null distribution are both normal, to decrease the information loss. Third, in this work, we focused on the S-genes with subtype-specificity or with functional relations reported to key pathways (e.g., MAP-kinase) or biological processes (e.g., EMT), and therefore the number of S-genes was limited. The limitation of scalability to a larger perturbation scale could be one future direction to improve our method. In our simulation study, greedy hill-climbing demonstrated high and robust performance in signaling networks with up to 30 S-genes, which meets the regular need for signaling network inference and drug targets prioritization. Many techniques may improve the performance of MCMC sampling (Andrieu et al., 2003), which warrants further exploration in our future work. Last but not least, we can also change the modeling framework radically using graph embedding based methods (Yue et al., 2020), as the observation of S-genes and E-genes are all high-dimensional vectors. However, the question of how to preserve the assumption of nested subset structures in the embedding space needs to be conquered tactfully.

In conclusion, NEM-Tar presents a useful computational framework for dissecting the regulatory architecture underlying specific cancer subtypes and prioritizing potential drug targets. With the explosive increase of high-throughput sequencing data, NEM-Tar warrants further evaluation using large-scale multi-omics data cohorts in the future.

## Data Availability Statement

The datasets analyzed during the current study are available in the TCGA repository (https://cancergenome.nih.gov/).

## Author Contributions

XW contributed to study concept and design. YZ and LZ contributed to data collection, analysis, and interpretation. XW contributed to critical revision of the manuscript for important intellectual content. LZ provided important advice and assistance for manuscript drafting. XW supervised the study. All authors read and approved the final manuscript.

## Conflict of Interest

The authors declare that the research was conducted in the absence of any commercial or financial relationships that could be construed as a potential conflict of interest.
