# Supplementary material for: NEM-Tar: A Probabilistic Graphical Model for Cancer Regulatory Network Inference and Prioritization of Potential Therapeutic Targets From Multi-Omics Data
Source: Front Genet. 2021 Apr 22;12:608042. doi: 10.3389/fgene.2021.608042 (PMC8100334; doi:10.3389/fgene.2021.608042)
Supplement: Supplementary file 1 [file Image_1.pdf]

MCMC sampling algorithm for NEM-Tar

Input : D, S*, G_{init}

for i=1→N, do

propose G* by randomly adding or deleting an edge with equal probabilities from all

gene pairs in the current graph G**(uniform jumping distribution)**

Compute the acceptance ratio

$$\alpha_G{}^{(i)} = \frac{P(G^{(i)} = G^* | S^*, D, \Theta)}{P(G^{(i)} = G | S^*, D, \Theta)} \bullet \frac{P(G|G^*)}{P(G^*|G)}$$

Draw $u_G{}^{(i)} \sim Uniform[0, 1]$

if $u_G{}^{(i)} < \alpha_G{}^{(i)}$,then

Set $G^{(i)} = G^*$

else

Set $G^{(i)} = G^{(i-1)}$

end if

end for

Compute the expected signal graph G from MCMC samples

$$E[G] = \frac{1}{N - N_b} \sum_{i=1}^{N-N_b} G^{(i)}$$

Figure S1. Metropolis-Hastings MCMC sampling algorithm for NEM-Tar.
